# Effect of Bioprocessing on Techno-Functional Properties of Climate-Resilient African Crops, Sorghum and Cowpea

**DOI:** 10.3390/foods11193049

**Published:** 2022-09-30

**Authors:** Markus Nikinmaa, Stefano Renzetti, Riikka Juvonen, Natalia Rosa-Sibakov, Martijn Noort, Emilia Nordlund

**Affiliations:** 1 VTT Technical Research Centre of Finland, P.O. Box 1000, FI-02044 Espoo, Finland; 2 Wageningen Food & Biobased Research, Wageningen University & Research, P.O. Box 17, 6700 AA Wageningen, The Netherlands

**Keywords:** sorghum, cowpea, bioprocessing, fermentation, enzymes, baking

## Abstract

Sorghum and cowpea are very compatible for intercropping in hot and dry environments, and they also have complementary nutritional compositions. Thus, the crops have the potential to improve food security in regions threatened by climate change. The aim of this study was to investigate different enzymes (carbohydrate-degrading, proteases and phytases) and lactic acid bacteria (LAB) fermentation to improve the techno-functional properties of sorghum and cowpea flours. Results show that sorghum carbohydrates were very resistant to hydrolysis induced by bioprocessing treatments. Most of the protease treatments resulted in low or moderate protein solubilization (from ca. 6.5% to 10%) in sorghum, while the pH adjustment to 8 followed by alkaline protease increased solubility to 40%. With cowpea, protease treatment combined with carbohydrate-degrading enzymes increased the solubility of proteins from 37% up to 61%. With regard to the techno-functional properties, LAB and amylase treatment decreased the sorghum peak paste viscosities (from 504 to 370 and 325 cPa, respectively), while LAB and chemical acidification increased cowpea viscosity (from 282 to 366 and 468 cPa, respectively). When the bioprocessed sorghum and cowpea were tested in breadmaking, only moderate effects were observed, suggesting that the modifications by enzymes and fermentation were not strong enough to improve breadmaking.

## 1. Introduction

Food security is a growing concern in the wake of climate change in sub-Saharan Africa. Wheat consumption in sub-Saharan Africa has been growing steadily, driven by the demand for convenient foods such as breads, particularly in urban areas. However, local production has not been able to keep pace with the growing demand, leaving countries in the region dependent on imports [1]. The warming climate may further reduce local cultivation; for example, Shew et al. [2] estimated wheat yield reductions in South Africa ranging from 8.5 to 28.5%, depending on the climate temperature rise. Therefore, promoting the cultivation of climate-resilient crops could improve food security and self-sufficiency, as well as support the local economy [3]. Two major indigenous African crops, sorghum (*Sorghum bicolor*) and cowpea (*Vigna unguiculata*), are well-adapted to high temperatures and dry conditions and agronomically highly compatible, providing opportunities for intercropping and reducing nutrient runoff and soil erosion. Cowpea is also able to improve nutrient and water availability for sorghum [4]. Cowpea is the most important grain legume in Africa, with a production of ca 8.6 million tonnes per year, while sorghum is the fifth most produced cereal in the world with an annual production of ca 60 million tonnes [5]. In addition to compatibility from an agricultural point of view, their nutritional properties also complement each other, due to their complementary essential amino acid profiles.

Traditionally, sorghum has been used for production of porridges and flatbreads such as kisra, but more recently also for processed foods such as beer and other malt-based beverages and couscous [6]. Cowpeas are used as whole boiled beans as such or in soups, as well as in akara, a cowpea fritter, and moin-moin, a paste made of steamed cowpeas [7]. However, the use and economical value of these crops is hampered by their limited technological functionalities for making processed foods, where they cannot compete with wheat. To increase the cultivation and food use of sorghum and cowpea, feasible processing technologies are needed to improve their potential to produce nutritious and appealing foods for rapidly urbanizing consumers, among other functions [3]. Different functionalities are needed depending on the food application. In beverage applications, high solubility, colloidal stability and often also emulsification properties are preferred. When it comes to baking, gas holding characteristics, viscoelastic properties and heat setting are important. Flour components should contribute to, or at least not interfere with, gas bubble formation and stability during baking. Furthermore, sufficient water-binding properties and flour pasting properties are important parameters. 

In sorghum, the main storage proteins, the kafirins, are highly water-insoluble [8] and are considered to have low functionality due to their encapsulation in protein bodies [9] and tight packing with starch in the native grains [10,11]. In addition, kafirins form crosslinks during heating, which reduce digestibility and may cause challenges in food formulations [12]. The dietary fibre (DF) in sorghum is largely composed of extensively substituted glucuronoarabinoxylans, which are insoluble and resistant to hydrolysis by enzymes [13]. Bioprocessing, via fermentation or enzymatic treatment, has been used to improve the functionality of sorghum when it comes to modifying both DF and proteins. Renzetti et al. [14] reviewed the effect of enzymatic modification on the functionality of proteins in gluten-free doughs. The improvement observed due to protein hydrolysis is attributed to increased gas holding and batter rheology, as well as modification of pasting properties. Fermentation has been observed to affect sorghum functionality in food systems, e.g., improvement in bread structure [10,15] attributed to protein hydrolysis, as well as release of starch granules from protein. In the study of Elkhalifa et al. [10], untreated proteins in the dough liquid formed aggregates in the bread crumb, while after fermentation only minor aggregation was visible. Both studies found a profound effect on starch gelation properties. On the other hand, Renzetti et al. [16] found that protease treatment had a negative effect on sorghum breads, while starch gel resistance to deformation was reduced. Regarding the sorghum carbohydrates, besides the highly packed starch, degradation of the main and very insoluble DF component in sorghum, glucuronoarabinoxylans, has proven challenging. It requires several complementary enzyme types to give xylanolytic enzymes access to the xylose backbone, and despite this, only limited solubilization has been achieved [13].

The main challenges of using cowpea in food applications are related to the hard-to-cook phenomenon, which occurs when beans are stored in high moisture and temperature, have antinutrient content (including phytic acid and trypsin inhibitors), the low functionality of proteins when compared to animal-based counterparts and a beany flavour. Several studies have examined the effect of germination or fermentation, although the main focus has mostly been on degrading the antinutrients. Uwaegbute et al. [17] found a reduction in antinutrient content and improvement in some structural characteristics of food products when germinating cowpea, although sensory attributes overall were negatively affected by germination. Giami [18] observed a reduction in water absorption capacity and an increase in oil absorption of both fermented and germinated cowpea, when compared to untreated flour. Both treatments also reduced foam stability, possibly due to degradation of proteins that contribute to foaming properties. The literature on enzymatic treatment of cowpea is limited. Segura-Campos et al. [19] examined the impact of enzymatic hydrolysis on selected functional properties of cowpea protein concentrate and found that protein solubility was increased near the isoelectric point by the treatments, and the surface hydrophobicity of the proteins was also increased. 

The aim of this study was to investigate the potential of bioprocessing via different carbohydrate- and protein-hydrolyzing enzyme treatments and lactic acid bacteria fermentations and their combinations to tailor the techno-functional properties of sorghum and cowpea flours. Specifically, pasting properties, carbohydrate solubility, water binding, protein solubility and DSC were analysed for the treated flours. Selected samples were tested further to evaluate the impact of the treatments in breadmaking. 

## 2. Materials and Methods

### 2.1. Raw Materials 

Sorghum flour (King Korn, Fine Mabele Flour, South Africa) and cowpea flour (variety: Bechuana white, South Africa) were used as raw materials for the bioprocessing treatments. Protein content was analysed with the Kjeldahl method (AOAC method 2001.11) using a Kjeldahl autoanalyser (Foss Tecator Ab, Höganäs, Sweden) nitrogen conversion factor of 6.25. Total, insoluble and soluble fibre was analysed with the AACC method 2011.25 using a semi-automated dietary fibre analyser (ANKOM TDF Fiber Analyzer, ANKOM, Macedon, NY, USA). Starch content was analysed according to AOAC Method 996.11 using a Megazyme Total Starch Assay kit (K-TSTA-100A, Megazyme, Wicklow, Bray, Ireland). 

### 2.2. Enzymes and Microbial Strains 

A variety of fibre-degrading enzyme preparations that included several activities, such as cellulase, arabinase, hemicellulose and xylanase, were tested to evaluate if the fibre components in sorghum could be solubilized via enzymatic processing. The DF-degrading enzymes tested were Viscozyme L (Novozymes), which included cellulase, arabinase, xylanase, betaglucanase, endoglucanase and polygalacturonase activities [20,21]; Celluclast BG (Novozymes), which has cellulase, endoglucanase, endoxylanase, endomannase and polygalacturonase activities [21]; Veron CP (AB Enzymes), which includes cellulase, xylanase, betaglucanase, polygalacturonase and endoglucanase [22]. Proteases tested were Brewer’s Clarity (Murphy & Son/DSM), a proline endopeptidase [23]; FlavourSEB NP (Advanced Enzymes), a leucine endopeptidase and endoprotease; Corolase 7089 (AB Enzymes), a serine endoprotease and metalloprotease; and Alcalase (Novozymes), an alkaline protease [24]). In addition to the DF- and protein-hydrolyzing enzymes, a phytase (Ultrabio) was used in the trials. Enzyme preparations were chosen based on their food-grade status, commercial availability and wide range of different activities on proteins and cell wall components. 

Strains of lactic acid bacteria that known to be able to ferment leguminous and cereal raw materials and potentially possess useful enzyme activities were selected from the VTT Culture Collection, including *Lactobacillus plantarum*, *Pediococcus pentosaceus* and *Leuconostoc pseudomesenteroides*. The *P. pentosaceus* strain has been previously found to degrade raffinose oligosaccharides, and has PepN activity and phytase and β-glucosidase activities, while *Leuconostoc pseudomesenetroides* produces dextran, which could have technological benefits in baking applications.

### 2.3. Bioprocessing Treatments 

Various bioprocessing treatments with different enzymes and starter cultures were performed, as summarized in Table 1. All enzyme treatments were performed for 4 h at 50 °C. Samples were mixed with 50 °C water and enzyme at the beginning of each treatment. Reactions were performed at 20% solids content. The pH of the Alcalase sample was adjusted to 8 with NaOH over the entire treatment, before being adjusted back to native pH (6.3) at the end of the treatment with HCl. A control sample without enzyme was also prepared, with the same pH adjustments and treatment time. 

LAB strains were revived from frozen stock cultures on de Man Rogosa Sharpe agar (MRS, Thermo Fisher Scientific Oxoid Ltd., Basingstoke, UK) plates for 72 h, followed by 24 h of anaerobic propagation in MRS broth (Thermo Fisher Scientific Oxoid Ltd., Basingstoke, UK). For the inoculation of the fermentations, the strains were finally cultivated in GEM broth for 24 h at 30 (General Edible Medium, containing 2% *w*/*v* glucose, 3% *w*/*v* soy peptone, 0.7% *w*/*v* yeast extract, 0.1% *w*/*v* MgSO_4_ in 0.01 M pH 6.3 potassium phosphate buffer). For inoculation of the water–flour suspensions, the cells were collected via centrifugation (4000× *g*, 15 min), washed once with sterile water and re-suspended in Milli-Q water. The aim was to have a starting cell density of 10^6^–10^7^ CFU/g. Fermentations were carried out statically at 30% solids content for 24 h at 30 °C, with addition of enzyme together with the starter cultures where applicable. 

All samples were frozen at −18 °C and freeze-dried (Christ Alpha 1–4, Martin Christ Gefriertrocknungsanlagen GmbH, Osterode am Harz, Germany) at the end of treatment. 

### 2.4. Chemical Characterisation of the Bioprocessed Samples

#### 2.4.1. Protein Analysis

Protein solubility was analysed in triplicate using a DC Protein assay kit (Bio-Rad Laboratories Inc., Hercules, CA, USA). A total of 1 g of flour was suspended in 9 mL of water, mixed carefully and then centrifuged. Solubility was calculated by dividing the soluble protein by the total protein in the sample, as analysed by Kjeldahl. 

#### 2.4.2. Carbohydrate Analysis

Soluble carbohydrate content and quality were analysed as earlier described [25]: (i) free sugars from the water extract, (ii) sugars (from hemicellulose and starch) after hydrolysis with 2 M trifluoracetic acid (TFA) for 1 h at 100 °C of water extract, and (iii) sugars from the other carbohydrates (mainly cellulose) were identified after sulfuric acid hydrolysis according to Seamaen et al. [26]. For preparation of the water extracts, about 1 g of sample was weighed in a 50 mL Greiner tube, and deionized water was added to reach 25 mL in volume. The solution was then kept under stirring for 2 h at room temperature. After stirring, the solution was centrifuged for 10 min at 16,000× *g*, and the supernatant was recovered. For the water-extractable arabinoxylans (TFA) contents, 1 mL of 2 M TFA was added to 1 mL of water extract. The obtained monosaccharides after each phase (i–iii) were determined via high-performance anion exchange chromatography (HPAEC) using an ICS-3000 Ion Chromatography HPLC system equipped with a CarboPac PA-1 column (250 × 2 mm^2^) in combination with a CarboPac PA guard column (25 × 2 mm^2^) and a pulsed electrochemical detector in pulsed amperometric detection mode (Dionex, Sunnyvale, CA, USA) at 20 °C according to Gilbert-lópez, Mendiola and Fontecha, 2015 [27]. Analyses were performed in duplicate. 

#### 2.4.3. Phytate Analysis

The phytic acid was analysed in selected samples (SEnzP2 + Ph, SLab + EnzMix, CEnzP1 + Ph and CLab + Enz) in triplicate according to a colorimetric method described by Vaintraub and Lapteva [28]

### 2.5. Microbiological Analyses

Microbiological analysis was performed for the fermented samples. Samples were serially diluted in Ringer’s solution (Merck), and 0.1 mL of dilutions was plated on various solid media. The viable counts of lactic acid bacteria (LAB) were enumerated on MRS agar in anaerobic conditions at 30 °C for 3–5 days. Aerobic heterotrophic bacteria were enumerated on a Difco^TM^ tryptic soya agar (TSA, BD Life Sciences, Franklin Lakes, NJ, USA) after incubation at 30 °C for 2–3 days. For determination of bacterial spores, the diluted sample was heated in a water bath at 80 °C for 10 min prior to plating on TSA plates. The bacterial growth media were supplemented with 0.001% cycloheximide (Sigma Chemical, St. Louis, MO, USA). Yeast and moulds were cultivated on Yeast Mould (YM) agar (BD Life Sciences) supplemented with chlortetracycline and chloramphenicol (both at 0.01%) and Triton-X 100 (0.02%, BDH) at 25 °C for 3–5 days. The microbial counts were expressed as colony-forming units per gram of the sample.

### 2.6. Moisture Sorption Behaviour of Native and Treated Flours

The moisture sorption behaviour of the different flour samples was determined in duplicate according to Erickson et al. [29] using an automatic multi-sample moisture sorption analyser SPSx-11l from Projekt Messtechnik (Ulm, Germany).

### 2.7. Thermal Analysis of Native and Treated Flours

Thermal analysis was performed with a TA Instruments type Q200 Modulated Differential Scanning Calorimeter (DSC) to measure starch gelatinization and protein denaturation. Gelatinization and denaturation were studied at flour concentrations (on dry matter basis) of 20% in distilled water.

A roughly 6 mg sample was weighed in stainless-steel cups, and water was added. Cups were then hermetically sealed and left to hydrate overnight. Samples were then analysed in a DSC, first by equilibrating them at −5 °C for 5 min and then heating them up to 160 °C at a rate of 5 °C/min. The onset of starch gelatinization and protein denaturation (Tonset), peak temperature (Tmin), end temperature (Tend) and gelatinization/denaturation enthalpy was determined using the analysis tool available in the Universal Analysis software. Experiments were performed in triplicate.

### 2.8. Rapid Viscous Analysis (RVA) of Native and Treated Flours

Pasting behaviour of all the samples was investigated using a Rapid Visco Analyser Super 4 (Perten, Hägersten, Stockholm, Sweden). Briefly, sample suspensions of 8% dry matter (dm) in water for a total weight of 25.0 g were used. Samples were subjected to a time–temperature profile. Initial stirring speed was 960 rpm at 50 °C for 60 s. Then, the stirring speed was decreased to 160 rpm while the temperature was increased to 95 °C within 3 min 42 s. Samples were then held at 95 °C for 2 min 30 s minutes and cooled to 50 °C within 3 min 48 s. Finally, samples were held at 50 °C for 2 min. Experiments were performed in duplicate. Data analysis was performed using TCW3 software (Perten, Hägersten, Stockholm, Sweden).

### 2.9. Water-Binding Capacity and Soluble Solids of Native and Treated Flours

The water-binding capacity (WBC) of the bran fractions was determined in triplicate according to a modified version of the protocol of Zanoletti et al. [30]. Flours (0.4 g on dry basis) were placed in 5 mL Eppendorf tubes, and 3.6 g of distilled water was added during vigorous stirring. After mixing on a vortex, the samples were left to shake at room temperature for 20 min on a Multi Reax Vortex from Heidolph (Schwabach, Germany). Then, the samples were centrifuged for 10 min at 5000× *g* using an Avanti J-26XP High-Speed Centrifuge from Beckman Coulter (Indianapolis, IN, USA). The supernatant was collected, and the pellet was drained for 15 min at an angle of 45° and then weighed. WBC was expressed as follows:

WBC = (wet pellet (g) − dried pellet (g))/(dried pellet (g))

For the determination of the soluble solids, the collected supernatant was dried overnight in an oven at 105C. The soluble solids were expressed as follows:

Soluble solids (g/g) = (dry weight of supernatants (g))/(dry weight of flour (g))

### 2.10. Assessment of Flour Functionality in Tin Bread Application

A reference bread dough formulation consisting of the climate-resistant crop flours sorghum, cassava and cowpea was used as recently developed [31]. The reference recipe consisted of 50 g of sorghum flour, 50 g of cassava flour, 9.5 g of cowpea flour, 8 g of psyllium husk powder, 5.5 g of dry yeast, 4 g of rapeseed oil, 4 g of sucrose, 2.5 g of salt and 119 g of water. From the reference recipe, the bioprocessed sorghum and cowpea flours were replaced on a one-to-one basis with the modified flours selected based on their techno-functionality. 

Bread preparation was performed as previously described [31]. In total, about 200 g of dry ingredients was added to a Brabender Farinograph (Brabender GmbH & Co. KG, Duisburg, Germany) and pre-mixed at a speed of 63 rpm. The mixing chamber was pre-set at 20 °C. Then, water was slowly added during mixing, which was performed for 6 min. After mixing, the dough was divided and shaped manually and put into three greased baking tins (158 mL volume; 10 cm × 4.5 cm × 3.5 cm). Each tin contained 105 g of dough. These tins were put in fermentation cabinets at 30 °C and 85% RH. The proofing time was defined as the time needed by 50 g of dough to reach a CO_2_ production of 90 mL. The CO_2_ production was determined using a Risograph (National Manufacturing, Lincoln, NE, USA). After proofing, the doughs were put in a swing oven at 180 °C for 40 min. During the first minute, steam was injected twice to regulate the moisture content. After baking, the breads were cooled at room temperature for 40 min, sealed in plastic low-density polyethylene bags and stored at room temperature until further analysis one day after baking. Three breads were baked for each variation.

### 2.11. Bread Quality Evaluation

Loaf volume was determined on 3 loaves, with a rapeseed displacement according to the AACC method 10–05.01. Specific volume (SV) was calculated as loaf volume divided by loaf weight (mL/g). 

Crumb texture was measured by means of texture profile analysis using a TA-XT2i Texture Analyser from Stable Micro Systems (Godalming, UK) with a 30 kg load cell and a 75 mm compression plate and performed as described by [30]. In total, 5 measurements were performed per bread type.

The moisture content of the bread crumbs (5 g sample) was measured according to the AACC standard method 44–15.02 by drying overnight in aluminium dishes in an oven at 105 °C. The filled dishes were cooled for 1 h in a desiccator before weight determination. In total, 3 measurements were performed per bread type.

### 2.12. Statistical Data Analysis

One-way ANOVA with Tukey’s HSD was performed to assess statistical significance between treatments using IBM SPSS 28 software. Principal component analysis and correlation analysis were performed with Rstudio (RStudio version 1.1.463, Inc., Boston, MA, USA).

## 3. Results and Discussion

### 3.1. Effects of Treatments on Chemical Characteristics

The raw materials, i.e., sorghum and cowpea flour, before any processing, contained 10.2 and 23.9% protein, 11.5 and 20.6% DF (of which 2.3 and 7.5% were soluble) and 73.1 and 40.7% starch, respectively. Solubility of protein and carbohydrates was higher in cowpea than in sorghum (Table 2 and Table 3).

Via bioprocessing of sorghum, the cellulolytic enzyme preparation Viscozyme L was found to be the most efficient when the levels of free sugars and solubilized carbohydrates were analysed (Table 2). The main increase in the analysed sugars was in the form of free glucose, which suggests the degradation of the cellulose and beta-glucan present in sorghum by beta-glucosidase activity of Viscozyme L. α-amylase (BAN480L) was not efficient at degrading starch in the chosen conditions either, showing lower contents of soluble sugars than the DF-degrading enzymes. Unexpectedly, the FlavourSEB protease preparation seemed to also have some carbohydrate-degrading activity, since sugar contents were also increased after that treatment. No xylose or arabinose was released, indicating that the enzymes were unable to degrade the glucuronoarabinoxylans, despite the various activities of the enzyme cocktails tested [20,32]. This is in accordance with Verbruggen et al. [13]), who showed that these polysaccharides are very resistant to enzymatic hydrolysis due to the large number of substitutions, including glucuronic acid and ferulic acid. Therefore, specific side-chain active enzymes, such as glucuronidases and ferulic acid esterases, would have been needed to facilitate the solubilization and degradation of sorghum glucuronoarabinoxylans. When the protein content was analysed, it was seen that the tested proteases as such managed to solubilize up to 10% of the total protein, with the Corolase enzyme preparation being the most efficient. However, when hydrolysis was carried out at pH 8 with Alcalase protease, 42% of the sorghum protein was solubilized, compared to ca. 8% with the same treatment time at pH 8 without the enzyme. Most probably, the alkaline protease was able to act on the proteins more efficiently, since typically, plant proteins are more soluble in alkaline conditions. This pH-driven solubilization was not detectable in the control pH 8 treatment, since the pH was reduced back to native and pH3 for the protein solubility analysis.

When individually inoculated into sorghum, *L. plantarum* and *P. pentosaceus* reached log 9 cfu/g at the end of fermentation, acidifying the material to pH 3.85–3.87. Dextran-producing *Lc. pseudomesenteroides* showed weaker growth and acidification capacity (pH 4.46). Co-fermentation of the sorghum dough with *L. plantarum* and *P. pentosaceus* led to a pH decrease to 3.96. Addition of the enzyme mixture enhanced the acidification, and pH 3.55 was reached after 24 h. Microbiological quality of the ferments was acceptable, in that the viable counts of fungi or aerobic bacteria remained low during the fermentations (<3.7 log cfu/g). Fermentations with LAB strains overall reduced both soluble protein and carbohydrate content, which can be explained by the microbes using these as a source of energy and nitrogen. A similar reduction in the soluble protein of sorghum due to fermentation has been observed in previous studies [10,33].

When bioprocessing was applied to cowpea, the biggest increase in soluble carbohydrate content was observed when α-amylase (BAN480L) was included in bioprocessing (4.2–4.6% free sugars; 12.6–13.9% total soluble sugars, Table 3), although cellulolytic Viscozyme L treatment in combination with protease FlavourSEB also increased the amount of sugars (4.4% free sugars; total soluble sugars 7.6%) compared to the control (6.7%). When protein solubilization was analysed, protease treatments were observed to increase the amount of soluble protein up to 60% (FlavourSEB samples) compared to 37% in the control at native pH (ca. 6.2). In acidic conditions (pH 3), phytase treatment together with the protease increased protein solubility from 16 to 44%. Phytase has also been previously shown to increase the solubility of legume proteins in acidic conditions [34]. It should be noted that the highest protein solubility (78.6%) was in the reference cowpea (pH 6.2) flour without any treatment. This indicates that the freeze-drying process needed for analytical purposes caused some protein aggregation, as the control sample (freeze-dried cowpea flour after 4 h treatment at 50 °C) had a protein solubility of 37%. Reduced solubility and denaturation caused by freeze drying has been observed previously for soy [35] and faba bean protein isolates, as well as pea globulin extracts [36], when compared to spray drying [37]. Yang et al. [38] observed similar solubility values in mild alkaline-extracted pea protein for both freeze-dried and spray-dried samples, as compared to the native proteins in this study, although the analysis was performed at pH 7, whereas the analysis pH in this study was 6.3. However, as all these studies were conducted on protein isolates with pH shifting, they are not entirely comparable to the whole flour used in this study.

Individual fermentation with *L. plantarum* or *Lc. pseudomesenteroides* acidified the cowpea to a pH value of 4.02–4.06. Acidification with *P. pentosaceus* was relatively weak (pH 4.65), and the final LAB count remained below 9 log cfu/g. Simultaneous fermentation using *L. plantarum* and *P. pentosaceus* dropped the pH value to 4.14 and 3.98 without and with added enzymes, respectively. Viable counts of aerobic bacteria and fungi remained constant during the fermentations. Fermentation treatments again reduced the amount of soluble carbohydrates and proteins, most likely again due to consumption of the amino acids and sugars by the microbes. Fermentation also showed increased free galactose levels, indicating that the fermentation reduced contents of galacto-oligosaccharides, which has also been observed previously. The *P. pentosaceus* strain used in this study was previously shown to reduce the raffino-oligosaccharide content of faba bean during fermentation [39].

Overall, total soluble solids reflected the differences observed in the soluble carbohydrate and protein contents in both crops; i.e., the samples where protein and/or polysaccharides were solubilized also had increased total soluble solid content (Table 2 and Table 3).

### 3.2. Effect of Treatments on Techno-Functional Properties

Flour pasting properties as measured by the Rapid Viscoanalyzer (RVA), i.e., how viscosity is affected during heating and cooling, can predict the behaviour of the material in several processes. In gluten-free baking, where starch is the main structure former, pasting properties play a vital role. In the present study, RVA viscosities in sorghum (peak, hold, final and set back) were all decreased by treatment with alpha-amylase and fermentation with *L. plantarum* + *P. pentosaceus* + Enzymes (SLab + EnzMix) (Table 4). Treatment of sorghum with the proteases Corolase and FlavourSEB (alone or combined with phytase) were the only ones able to significantly increase the final viscosity (>1000 cP) compared to the sorghum control (952 cP). Pasting temperature was increased after treatment with FlavourSEB + Viscozyme (94.6 °C, compared to 93.5 °C in the sorghum control). The changes described above are significantly different from the sorghum control and sorghum raw material. However, the changes observed were not very intense (Table 4). The main difference was observed in the breakdown viscosity, as the sorghum control had 10 cP, while the treatment with Lab combined with Enzymes (SLab + EnzMix) reached 94.5 cP. Alcalase treatment also modified some of the pasting properties of sorghum (i.e., increased peak and breakdown, decreased final and set back viscosity), but the alkaline reference treatment of sorghum had a similar impact, making it difficult to understand if the changes are linked to the solubilization of protein by Alcalase or due to other changes by the pH shift. On the other hand, Chew-Guevara et al. [40] observed a similar effect, where peak viscosity increased but final viscosity decreased after decortication and protease treatment of sorghum. Fermentation of cowpea showed an opposite effect on RVA viscosities compared to sorghum, as a significant increase in the peak, hold and breakdown viscosities was observed (Table 5). An even more pronounced increase in viscosity values was detected by chemical acidification; thus, the viscosity increase is linked to acidification of the material. A fermentation-mediated viscosity increase was also reported by Lu and Sanni-Osomo [41] in cowpea and in yellow pea by Li et al. [42], who suggested the changes in the starch granule behaviour played a role in the changes. In cowpea, amylase almost completely removed the viscosity effect of starch on heating, indicating the enzyme had better access to starch granules than in sorghum. 

In addition to pasting properties, the interaction of the flour with water (e.g., water-binding capacity and moisture sorption) is important in gluten-free baking as it influences dough consistency as additional ingredients, such as hydrocolloids, are often added to confer these properties [43]. In both sorghum and cowpea, moisture sorption at 0.95 a_w_ g water/g dm increased with an increased content of soluble carbohydrates (Table 4 and Table 5). Soluble components, such as sugars, show a rapid increase in moisture sorption when water activity increases [44]. Similar behaviour has been found in malted sorghum, where starch degradation led to higher moisture sorption at high water activity [45]. The WBC of sorghum was significantly higher (compared to both control and reference samples) only in the alkaline reference, while for cowpea, the treatments did not affect, or decreased the WBC compared to the control (Table 4 and Table 5). Generally, the WBC of sorghum was lower than that of cowpea. 

Concerning the thermal analysis, for sorghum, onset temperature was not significantly affected (only SLab2 decreased it), and peak temperature was higher after Alcalase, alkaline reference and SLab + EnzMix treatments. Previously, Chew-Guevara et al. [40] observed an increase in DSC Tonset and Tpeak after protease treatment of sorghum. In cowpea, onset temperature was higher in the fermented samples (CLab1 + L2 and CLab + Enz), while most of the enzyme treatments increased the peak temperature. However, the changes were rather moderate. 

### 3.3. Principal Component Analysis

**Figure 1:**
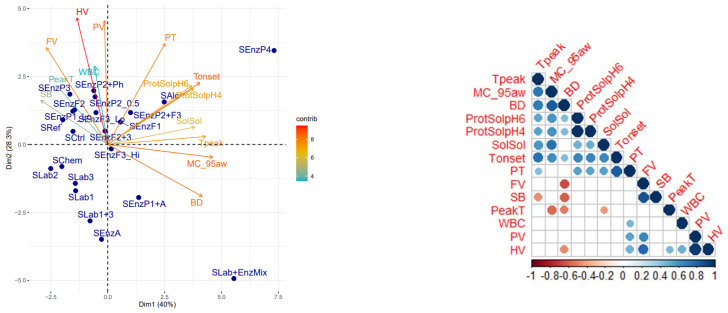
PCA (**left**) and correlation analysis (**right**) between measured parameters of sorghum. Sorghum sample codes are explained in Table 1. Measured parameters: Tpeak = peak temperature at which biopolymers melted, Tonset = onset temperature at which biopolymers began melting, WBC = water-binding capacity, MC_95 aw = moisture sorption at 0.95 aw, SolSol = soluble solids, PV = peak viscosity in RVA, HV = hold viscosity in RVA, FV = final viscosity in RVA, SB = set back viscosity, BD = breakdown viscosity, PT = pasting temperature in RVA.

A principal component analysis (PCA) was performed to gain further insights into the effect of the treatments on the physicochemical properties of sorghum flour. The first two principal components (i.e., PC_1_ and PC_2_) explained 68.3% of the variance, thus providing a good representation of the differences among samples (Figure 1). Variations among PC_1_ could be mainly associated with starch melting temperature, flour moisture sorption and soluble components (solids and proteins). Variations in PC_2_ were associated with pasting properties of the flour. In general, most of the treatments increased starch melting temperature, moisture sorption and the solubilization of solids, including proteins compared to the untreated sorghum. Pasting behaviour was most largely affected by treatments with lactic acid bacteria and α-amylases and combinations there off, resulting in decreased paste viscosities. Alkaline protease treatment (SEnzP4) and LAB treatment combined with enzymes (SLab + EnzMix) provided the largest changes in the physicochemical properties of sorghum. Alkaline treatments were linked to increased protein solubility and starch melting temperature, while the pasting properties were only slightly affected. On the contrary, sample SLab + EnzMix showed a concomitant increase in protein solubility and starch melting temperature and a significant reduction in paste viscosities, which were largely associated with the LAB activities.

Significant positive correlation between the Tonset of starch gelatinization and PT were observed. Both characteristics are related to how starch behaves during heating; Tonset describes the temperature at which starch granules start melting, while PT describes the temperature at which the viscosity of the starch-water mixture starts to rise during the RVA measurement due to starch swelling. Soluble solids correlated with Tpeak, i.e., the peak temperature of starch gelatinization. The observed increase in starch gelatinization temperature may be well associated with the release of free sugars and soluble fibres which reduce the plasticizing ability of the water phase [46]. 

**Figure 2:**
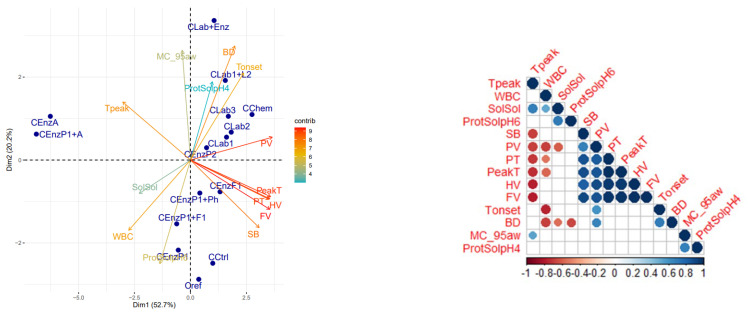
PCA (**left**) and correlation analysis (**right**) between measured parameters of cowpea. Cowpea sample codes are explained in Table 1. Measured parameters: Tpeak = Biopolymers melting, peak temperature, Tonset = Biopolymers melting, onset temperature, WBC = water-binding capacity, MC_95 aw = Moisture sorption at 0.95 aw, SolSol = soluble solids, PV = peak viscosity in RVA, HV = hold viscosity in RVA, FV = final viscosity in RVA, SB = Set back viscosity, BD = breakdown viscosity, PT = Pasting temperature in RVA, ProtSolpH6 = protein solublity at pH 6, ProtSolpH4 = protein solublity at pH 4.

In the principal component analysis (PCA) of cowpea, the first two principal components (PC) of the cowpea PC explained 72.9% of the variation in the data (Figure 2). Variations among the PC_1_ were associated with pasting properties of the flour. Variations among the PC_2_ could be mainly associated with starch melting temperature, flour moisture sorption and soluble proteins. All treatments showed to increase moisture sorption, soluble proteins (at pH 4) and the onset temperature of starch gelatinization compared to the untreated cowpea. While treatments generally enhanced paste viscosities, amylase-treated samples were the only ones to show a drastic reduction in viscosity parameters. LAB and chemical acidification treatments were linked to high peak viscosity values, but also to higher protein solubility in pH4. It has been earlier reported that in cowpea the starch is tightly covered with protein material [47,48] and changes in the protein structures significantly affects pasting behaviour [31]. Hence, weakening of the protein barrier around starch granules, as suggested by increased solubilization, results in increased starch swelling and consequently higher paste viscosities. Overall, correlations between sorption data, soluble solids and melting temperatures were similar to those observed for sorghum.

### 3.4. Breadmaking

The functionality of the selected modified sorghum and cowpea flours was assessed in baking trials. For such a purpose, the native sorghum flour was replaced one to one with the freeze-dried bioprocessed flours. The rest of the reference formulation was kept the same. Next, the same approach was used to replace the cowpea flour with bioprocessed flours. Significant effects (*p* < 0.05) were observed in terms of the specific volume of bread and crumb properties (i.e., moisture content and instrumental texture) (Table 6). When compared to the control sample (SCtrl), alkaline protease treatment (SEnzP4) of sorghum increased the bread volume, but the difference was not significant when compared to the reference bread. Compared to the reference bread, only the sample treated with protease and amylase (CEnzP1 + A) showed a significant increase in specific volume, while all other formulations showed similar values. However, it should be noted that this same sample, CEnzP1 + A, resulted in a large hole in the middle of the breadcrumbs. In the RVA, these sorghum flours showed a general reduction in paste viscosities, likely due to the action of α-amylase. Considering that the freeze-dried flours were not heat-treated, it is likely that the enzymatic action also affected the cassava and cowpea starches during proofing and in the early stages of baking. Consequently, a further reduction in the paste viscosity of the flour mixture resulted in the observed structural collapse in the crumb during baking. 

Crumb moisture was significantly affected by the bioprocessing treatments (*p* < 0.05). All the modified sorghum flours resulted in a significant reduction in crumb moisture compared to the reference. Among the modified cowpea flours, the protease- and amylase-treated sample (CEnzP1 + A) and LAB-fermented sample (CLab1 + L2) showed significantly higher moisture values than the reference. Crumb hardness was either significantly increased by the addition of the modified flours or not significantly affected. Overall, the reference crumb was among the samples with the lowest hardness. Crumb hardness was mainly controlled by crumb density, as indicated by the high correlation (R^2^ = 0.956, *p* < 0.05). Improved bread quality, especially improved shelf life, via LAB fermentation has been previously reported [49], but in the present study, the crumb properties were not measured as a function of storage time. Crumb chewiness was significantly lower for enzyme-treated samples SEnzP4 and CEnzP1 + A compared to the reference, while all other modified flours showed either an increase or no significant effects. Fermented samples SLab + EnzMix and CLab1 + L2 showed a significant increase in both crumb cohesiveness and resilience compared to the reference. Samples SAlc, CCrtl, CEnzP1 + F1, CLab1 + L2 and CChem showed a significant increase in resilience only. Springiness was not majorly affected by the modified flours, except for a significant reduction for sample CEnzP1 + A.

## 4. Conclusions

The bioprocessing of sorghum and cowpea with enzymes and microbes resulted in distinct modification in the techno-functional properties of the raw materials. Sorghum was rather resistant to bioprocessing: only alkaline enzyme treatment showed significant solubilization of proteins, whereas among carbohydrates, glucuronoarabinoxylan and starch were not significantly affected, but mostly cellulose and beta-glucan seemed to be hydrolyzed. Specific LAB and amylase treatments still caused some changes in the RVA profiles of the sorghum sample by decreasing the viscosity profiles. Cowpea was easier to attack—especially for proteases—and protein solubilization was notable. Acidification of cowpea by chemical means or LAB increased the pasting viscosity. However, when the selected samples were tested in gluten-free baking, the changes in bread quality were rather moderate. More severe treatments should probably be applied to break down the insoluble and complexed material, especially those with sorghum. Furthermore, it is worth testing the bioprocessed samples in other food applications, since techno-functional properties were still changed, and thus, the differences might be notable for other bakery and food applications. Additionally, to establish a commercially feasible process, the efficacy of bioprocessing should be evaluated without freeze drying of the ingredients before its use in baking or other applications.

## Figures and Tables

**Table 1:** Information on the enzymes and starter cultures used in bioprocessing treatments.

Sample Code	Crop	Enzyme(s) and Microbes Tested	Info on Bioprocessing Method	Enzyme Dosage (%)
SRef	Sorghum	None	Raw material	-
SCtrl	Sorghum	None	Control for 4 h 50 °C treatment	-
SEnzP1_Hi	Sorghum	Brewer’s Clarex	Protease	0.036
SEnzP1_Lo	Sorghum	Brewer’s Clarex	Protease	0.01
SEnzP2_0.5	Sorghum	FlavourSEB	Protease	0.5
SEnzP3	Sorghum	Corolase	Protease	1
SEnzP4	Sorghum	Alcalase	Alkaline protease	1
SAlc	Sorghum	Alkaline control	Alkaline control	-
SEnzF1	Sorghum	Celluclast BG	Fibre degrading	1
SEnzF2	Sorghum	Veron CP	Fibre degrading	1
SEnzF3_Lo	Sorghum	Viscozyme L	Fibre degrading	0.1
SEnzF3_Hi	Sorghum	Viscozyme L	Fibre degrading	1
SEnzF2 + 3	Sorghum	Veron CP + Viscozyme L	Fibre degrading	1 + 1
SEnzA	Sorghum	BAN480L	α-Amylase	0.2
SEnzP2 + F3	Sorghum	FlavourSEB + Viscozyme	Protease + Fibre degrading	0.5 + 1
SEnzP2 + Ph	Sorghum	Flavourseb + Ultrabio phytase	Protease + Phytase	0.5 + 1
SEnzP1 + A	Sorghum	Flavourseb + BAN480L	Protease + α-Amylase	0.5 + 0.2
SChem	Sorghum	Chemical acidification	Control for acidic conditions	-
SLab1	Sorghum	*L. plantarum*	LAB fermentation	-
SLab2	Sorghum	*L. pseudomesenteroides*	LAB fermentation	-
SLab3	Sorghum	*P. pentosaceus*	LAB fermentation	-
SLab1 + 3	Sorghum	*L. plantarum* + *P. pentosaceus*	LAB fermentation	-
SLab + EnzMix	Sorghum	*L. plantarum* + *P. pentosaceus* + Viscozyme + Ultrabio + Corolase	LAB fermentation + Fibre degrading + Phytase + Protease	1 + 1 + 1
CRef	Cowpea	None	Control (no treatment)	-
CCtrl	Cowpea	None	Control for 4 h 50 °C treatment	-
CEnzP1	Cowpea	FlavourSEB	Protease	0.5
CEnzP2	Cowpea	Corolase	Protease	1
CEnzA	Cowpea	BAN480L	Amylase	0.2
CEnzF1	Cowpea	Viscozyme	Fibre degrading	1%
CEnzP1 + F1	Cowpea	FlavourSEB + Viscozyme	Protease + Fibre degrading	0.5 + 1
CEnzP1 + Ph	Cowpea	FlavourSEB + Ultrabio Phytase	Protease + Phytase	0.5 + 1
CEnzP1 + A	Cowpea	FlavourSEB + BAN480L	Protease + Amylase	0.5 + 0.2
CChem	Cowpea	Chemically acidified	Control for acidic conditions	-
CLab1	Cowpea	*L. plantarum*	LAB fermentation	-
CLab2	Cowpea	*L. pseudomesenteroides*	LAB fermentation	-
CLab3	Cowpea	*P. pentosaceus*	LAB fermentation	-
CLab1 + L2	Cowpea	*L. plantarum* + *P. pentosaceus*	LAB fermentation	-
CLab + Enz	Cowpea	*L. plantarum* + *P. pentosaceus* + Viscozyme + UltraBio + Corolase	LAB fermentation + Fibre degrading + Phytase + Protease	1 + 1 + 1

**Table 2:** Effects of bioprocessing treatments on soluble carbohydrates and protein of sorghum. na = not analysed. Sample codes shown in Table 1.

	Free Sugars (g/100 g)			TFA Sugars (g/100 g)				H_2_SO_4_ Hydrolyzed Sugars (g/100 g)			Protein Solubility (% of Total Protein)	
Sample Code	Glucose	Fructose	Total FS	Galactose	Glucose	Total TFA	TFA-FS	Glucose	Total SO4	SO4-TFA	pH (Native) 6.3	pH 3
SRef	0.67 ^bc^	0.55 ^de^	1.21 ^bc^	<0.1 ^a^	0.95 ^bc^	0.95 ^bc^	−0.27 ^a^	0.95 ^b^	0.95 ^b^	0.00 ^ab^	6.86 ^cdef^	6.3 ^cd^
SCtrl	0.68 ^bc^	0.51 ^bc^	1.19 ^b^	<0.1 ^a^	0.91 ^b^	0.91 ^b^	−0.28 ^a^	0.97 ^b^	0.97 ^b^	0.06 ^ab^	6.24 ^abcde^	6.39 ^de^
SEnzP1_Hi	0.66 ^b^	0.53 ^bcd^	1.19 ^b^	0.102 ^b^	1.00 ^bcd^	1.11 ^cd^	−0.08 ^abc^	0.98 ^b^	0.98 ^b^	−0.13 ^ab^	7.06 ^def^	6.63 ^defg^
SEnzP1_Lo	0.76 ^c^	0.56 ^def^	1.32 ^c^	0.11 ^bc^	1.17 ^d^	1.28 ^d^	−0.04 ^bcd^	1.00 ^b^	1.00 ^b^	−0.28 ^a^	7.14 ^def^	6.93 ^defg^
SEnzP2_0.5	1.58 ^g^	0.57 ^ef^	2.15 ^f^	<0.1 ^a^	2.68 ^h^	2.68 ^h^	0.52 ^g^	3.04 ^e^	3.04 ^e^	0.36 ^bc^	8.55 ^ghi^	8.91 ^j^
SEnzP3	na	na	na	na	na	na	na	na	na	na	9.68 ^ijk^	9.61 ^kl^
SEnzP4	na	na	na	na	na	na	na	na	na	na	42.71 ^l^	42.17 ^n^
SAlc	na	na	na	na	na	na	na	na	na	na	8.08 ^fgh^	6.81 ^defg^
SEnzF1	1.27 ^f^	0.51 ^bc^	1.78 ^e^	0.12 ^c^	2.00 ^g^	2.12 ^g^	0.34 f^g^	2.01 ^d^	2.01 ^d^	−0.11 ^ab^	10.93 ^k^	10.12 ^l^
SEnzF2	0.95 ^de^	0.53 ^bcd^	1.48 ^d^	0.11 ^bc^	1.49 ^e^	1.61 ^e^	0.13 ^de^	1.39 ^bc^	1.39 ^bc^	−0.21 ^a^	7.39 ^efg^	6.93 ^defg^
SEnzF3_Lo	1.26 ^f^	0.54 ^cde^	1.79 ^e^	0.11 ^bc^	1.79 ^f^	1.90 ^f^	0.10 ^cde^	1.68 ^cd^	1.68 ^cd^	−0.22 ^a^	7.21 ^def^	7.05 ^fgh^
SEnzF3_Hi	2.39 ^j^	0.64 ^g^	3.03 ^h^	0.11 ^bc^	3.07 ^ij^	3.18 ^j^	0.15 ^def^	3.01 ^e^	3.01 ^e^	−0.17 ^a^	7.37 ^efg^	7.24 ^ghi^
SEnzF2 + 3	2.42 ^j^	0.59 ^f^	3.01 ^h^	0.11 ^bc^	3.12 ^ij^	3.24 ^j^	0.22 ^ef^	3.34 ^ef^	3.34 ^ef^	0.11 ^ab^	7.93 ^fgh^	7.67 ^hi^
SEnzA	0.98 ^e^	0.50 ^b^	1.48 ^d^	<0.1 ^a^	1.56 ^ef^	1.56 ^e^	0.08 ^cde^	1.73 ^cd^	1.73 ^cd^	0.17 ^abc^	6.6 ^bcde^	7 ^efg^
SEnzP2 + F3	2.20 ^i^	0.75 ^i^	2.92 ^h^	0.10 ^b^	4.18 ^k^	4.28 ^k^	1.36 ^i^	4.93 ^g^	4.93 ^g^	0.65 ^c^	7.49 ^efg^	7.74 ^i^
SEnzP2 + Ph	1.62 ^gh^	0.54 ^cde^	2.15 ^f^	<0.1 ^a^	2.98 ^i^	2.99 ^i^	0.83 ^h^	3.05 ^e^	3.05 ^e^	0.06 ^ab^	8.94 ^hij^	9.37 ^jk^
SEnzP1 + A	1.68 ^h^	0.67 ^h^	2.35 ^g^	0.10 ^b^	3.25 ^j^	3.35 ^j^	1.00 ^h^	3.57 ^f^	3.57 ^f^	0.22 ^abc^	6.02 ^abcd^	6.48 ^def^
SChem	0.86 ^d^	0.59 ^f^	1.45 ^d^	0.10 ^b^	1.13 ^cd^	1.23 ^d^	−0.22 ^ab^	1.20 ^b^	1.20 ^b^	−0.03 ^ab^	7.47 ^efg^	7.07 ^fgh^
SLab1	<0.1 ^a^	<0.1 ^a^	0 ^a^	<0.1 ^a^	0.14 ^a^	0.14 ^a^	0.14 ^def^	0.16 ^a^	0.16 ^a^	0.02 ^ab^	5.69 ^abc^	5.57 ^b^
SLab2	<0.1 ^a^	<0.1 ^a^	0 ^a^	<0.1 ^a^	0.11 ^a^	0.11 ^a^	0.11 ^cde^	0.13 ^a^	0.13 ^a^	0.02 ^ab^	6.48 ^bcde^	5.68 ^bc^
SLab3	<0.1 ^a^	<0.1 ^a^	0 ^a^	<0.1 ^a^	0.15 ^a^	0.15 ^a^	0.15 ^def^	0.17 ^a^	0.17 ^a^	0.02 ^ab^	5.51 ^ab^	5.19 ^b^
SLab1 + 3	na	na	na	na	na	na	na	na	na	na	5.01 ^a^	4.69 ^a^
SLab + EnzMix	na	na	na	na	na	na	na	na	na	na	10.14 ^jk^	10.87 ^m^

TFA and H_2_SO_4_ hydrolyzed sugars show content of water-soluble polysaccharides hydrolyzed by the respective acids. Letters denote homogenous subsets in ANOVA analysis.

**Table 3:** Effects of bioprocessing treatments on soluble carbohydrates and protein of bioprocessed cowpea samples. Sample codes shown in Table 1.

Sample Code		Free Sugars (g/100 g)					H_2_SO_4_ Hydrolyzed Sugars (g/100 g)				Protein Solubility (% of Total Protein)	
	Arabinose	Galactose	Glucose	Fructose	Sucrose	Total FS	Arabinose	Galactose	Glucose	Total SO_4_	pH (Native) 6.3	pH 4
CRef	<0.1 ^a^	0.57 ^a^	0.1 ^a^	0.78 ^c^	1.28 ^b^	2.93 ^b^	0.1 ^abc^	2.73 ^a^	2.45 ^b^	5.68 ^bc^	78.62 ^e^	15.80 ^a^
CCtrl	<0.1 ^a^	0.58 ^a^	1.24 ^e^	1.72 ^ef^	<0.1 ^a^	3.54 ^d^	0.1 ^ab^	2.89 ^a^	3.82 ^cd^	6.71 ^cd^	37.30 ^c^	15.80 ^a^
CEnzP1	<0.1 ^a^	0.58 ^a^	1.60 ^f^	1.72 ^ef^	<0.1 ^a^	3.89 ^e^	<0.1 ^a^	2.88 ^a^	4.50 ^cde^	7.38 ^cd^	58.97 ^e^	21.50 ^bcd^
CEnzP2	na	na	na	na	<0.1 ^a^	na	na	na	na	na	51.93 ^d^	40.33 ^g^
CEnzA	<0.1 ^a^	0.56 ^a^	1.81 ^h^	1.80 ^g^	<0.1 ^a^	4.16 ^f^	0.33 ^e^	2.65 ^a^	10.92 ^g^	13.90 ^e^	34.38 ^de^	17.28 ^ab^
CEnzF1	na	na	na	na	<0.1 ^a^	na	na	na	na	na	32.45 ^abc^	18.3 ^abc^
CEnzP1 + F1	<0.1 ^a^	0.63 ^b^	2.01 ^i^	1.75 ^fg^	<0.1 ^a^	4.40 ^g^	<0.1 ^a^	2.62 ^a^	4.98 ^e^	7.60 ^d^	60.38 ^e^	22.08 ^cd^
CEnzP1 + Ph	<0.1 ^a^	0.58 ^a^	1.69 ^g^	1.73 ^efg^	<0.1 ^a^	4.01 ^e^	<0.1 ^a^	2.85 ^a^	4.65 ^de^	7.50 ^d^	48.57 ^d^	43.84 ^g^
CEnzP1 + A	<0.1 ^a^	0.58 ^ab^	2.26 ^j^	1.72 ^ef^	<0.1 ^a^	4.56 ^h^	0.28 ^de^	2.54 ^a^	9.82 ^f^	12.64 ^e^	60.68 ^e^	22.90 ^de^
CChem	0.16 ^d^	1.16 ^c^	1.59 ^f^	1.66 ^e^	<0.1 ^a^	4.57 ^h^	0.16 ^bcd^	2.86 ^a^	3.53 ^c^	6.55 ^cd^	32.42 ^abc^	29.02 ^f^
CLab1	0.17 ^d^	1.14 ^c^	0.39 ^c^	0.23 ^a^	<0.1 ^a^	1.93 ^a^	0.18 ^cd^	2.69 ^a^	1.28 ^a^	4.15 ^ab^	29.16 ^ab^	24.30 ^de^
CLab2	0.11 ^b^	1.14 ^c^	0.27 ^b^	0.38 ^b^	<0.1 ^a^	1.89 ^a^	0.11 ^abc^	2.26 ^a^	0.91 ^a^	3.28 ^a^	30.58 ^ab^	26.94 ^ef^
CLab3	0.13 ^c^	1.18 ^c^	0.44 ^d^	1.35 ^d^	<0.1 ^a^	3.10 ^c^	<0.1 ^a^	2.57 ^a^	1.40 ^a^	3.97 ^ab^	27.58 ^a^	24.36 ^de^
CLab1 + L2	na	na	na	na	<0.1 ^a^	na	na	na	na	na	26.74 ^a^	24.43 ^de^
CLab + Enz	na	na	na	na	<0.1 ^a^	na	na	na	na	na	31.53 ^abc^	44.06 ^g^

H_2_SO_4_ hydrolyzed sugars show content of water-soluble polysaccharides hydrolyzed by H_2_SO_4_. Letters denote homogenous subsets in ANOVA analysis. na = not analyzed.

**Table 4:** Effect of bioprocessing treatments on techno-functional properties of sorghum flours. Sample codes shown in Table 1.

	Biopolymers’ Melting Temperature (DSC)		WBC	MC Sorption at 0.95 aw	Soluble Solids	Viscosity (cP)					Tpasting
Sample Code	Onset °C	Peak °C	g Water/g dm Pellet	g Water/g dm	(% dm)	Peak	Hold	Final	Set Back	Breakdown	°C
SCtrl	65.0 ^f^	73.8 ^abcde^	1.87 ^abcd^	0.28	3.2 ^abcd^	504 ^def^	494 ^defg^	952 ^j^	458 ^hi^	10 ^ab^	93.5 ^cd^
SEnzP1_Hi	64.6 ^cdef^	73.5 ^abc^	2.11 ^hi^	0.28	3.4 ^bcd^	569.5 ^jk^	563 ^i^	912 ^hij^	349 ^ef^	6.5 ^ab^	92.2 ^ab^
SEnzP1_Lo	64.6 ^cdef^	73.5 ^abc^	2.10 ^hi^	0.28	3.5 ^bcde^	558.5 ^hijk^	551.5 ^hi^	891.5 ^ghi^	340 ^ef^	7 ^ab^	93.5 ^cd^
SEnzP2_0.5	65.1 ^f^	73.8 ^abcde^	1.83 ^ab^	0.30	5.0 ^ghi^	571.5 ^k^	562 ^i^	1043.5 ^k^	481.5 ^i^	9.5 ^ab^	93.9 ^de^
SEnzP3	64.6 ^cdef^	73.3 ^a^	2.04 ^gh^	0.32	3.1 ^abcd^	567.5 ^jk^	561 ^i^	1134 ^l^	573 ^j^	6.5 ^ab^	93.7 ^cd^
SEnzP4	64.2 ^abcde^	74.8 ^h^	1.89 ^abcdef^	0.39	6.0 ^ij^	612 ^l^	545.5 ^hi^	764 ^cd^	218.5 ^b^	66.5 ^h^	95.5 ^f^
SAlc	65.2 ^f^	74.6 ^fgh^	2.18 ^i^	0.34	3.3 ^abcde^	625 ^l^	567 ^i^	733.5 ^c^	166.5 ^a^	58 ^h^	94.6 ^ef^
SEnzF1	65.2 ^f^	74.3 ^efgh^	2.02 ^fgh^	0.30	4.6 ^fgh^	507 ^def^	495 ^defg^	848 ^efg^	353 ^ef^	12 ^abc^	93.7 ^cd^
SEnzF2	64.6 ^cdef^	73.8 ^abcde^	1.99 ^defgh^	0.28	3.5 ^cdef^	563.5 ^ijk^	560 ^i^	944.5 ^ij^	384.5 ^fg^	3.5 ^a^	93.5 ^cd^
SEnzF3_Lo	65.1 ^f^	74.0 ^bcdef^	2.04 ^gh^	0.29	3.9 ^defg^	525 ^efghi^	517 ^fgh^	866.5 ^fgh^	349.5 ^ef^	8 ^ab^	93.5 ^cd^
SEnzF3_Hi	64.9 ^def^	73.7 ^abcde^	1.98 ^defgh^	0.31	4.7 ^gh^	491 ^def^	481.5 ^def^	799.5 ^de^	318 ^de^	9.5 ^ab^	93.9 ^de^
SEnzF2 + 3	64.9 ^ef^	73.8 ^abcde^	1.98 ^defgh^	0.30	4.7 ^gh^	489 ^de^	481 ^de^	821.5 ^ef^	340.5 ^ef^	8 ^ab^	93.9 ^de^
SEnzA	64.8 ^def^	73.7 ^abcde^	1.88 ^abcde^	0.28	4.4 ^efg^	325.5 ^a^	311.5 ^b^	583 ^ab^	271.5 ^cd^	14 ^bcd^	92.5 ^b^
SEnzP2 + F3	65.1 ^f^	74.2 ^defgh^	1.85 ^abcd^	0.33	6.6 ^j^	529.5 ^fghij^	520.5 ^gh^	864 ^fgh^	343.5 ^ef^	9 ^ab^	94.6 ^ef^
SEnzP2 + Ph	65.2 ^f^	74.4 ^efgh^	1.81 ^ab^	0.30	4.8 ^gh^	550.5 ^ghijk^	544 ^h^	1115.5 ^l^	571.5 ^j^	6.5 ^ab^	93.5 ^cd^
SEnzP1 + A	65.0 ^f^	74.1 ^cdefg^	1.93 ^bcdefg^	0.31	5.7 ^hij^	425 ^c^	392 ^c^	636 ^b^	244 ^bc^	33 ^fg^	92.8 ^bc^
SChem	63.8 ^ab^	73.6 ^abcd^	1.96 ^cdefg^	0.30	3.2 ^abcd^	482.5 ^d^	462 ^d^	881 ^gh^	419 ^gh^	20.5 ^cde^	91.5 ^a^
SLab1	64.2 ^abcd^	73.9 ^abcde^	1.82 ^ab^	0.28	2.4 ^ab^	514 ^defg^	485 ^defg^	849 ^efg^	364 ^ef^	29 ^ef^	91.4 ^a^
SLab2	63.8 ^a^	73.7 ^abcd^	1.86 ^abcd^	0.28	2.3 ^a^	523 ^efgh^	516.5 ^efgh^	874 ^fgh^	357.5 ^ef^	6.5 ^ab^	91.4 ^a^
SLab3	64.0 ^abc^	73.9 ^abcde^	1.93 ^bcdefg^	0.28	2.7 ^abc^	497.5 ^def^	474 ^d^	802.5 ^de^	328.5 ^e^	23.5 ^def^	92.3 ^ab^
SLab1 + 3	64.2 ^abcde^	73.7 ^abcde^	1.80 ^ab^	0.30	2.7 ^abc^	438 ^c^	398 ^c^	724.5 ^c^	326.5 ^e^	40 ^g^	92.2 ^ab^
SLab + EnzMix	64.0 ^abc^	74.7 ^gh^	1.76 ^a^	0.42	6.6 ^j^	370 ^b^	275.5 ^a^	528.5 ^a^	253 ^bc^	94.5 ^i^	92.2 ^ab^

Letters denote homogenous subsets in ANOVA analysis.

**Table 5:** Effect of treatments on techno-functional properties of cowpea. Sample codes shown in Table 1.

	Biopolymers’ Melting Temperature (DSC)		WBC	MC Sorption at 0.95 aw	Soluble Solids	Viscosity (cP)					Tpasting
Sample Code	Onset °C	T Peak °C	g Water/g dm Pellet	g Water/g dm	(% dm)	Peak	Hold	Final	Set Back	Breakdown	°C
CRef	67.0 ^bcde^	78.5 ^abc^	3.21 ^c^		17 ^abc^	219 ^b^	215 ^bc^	362 ^ef^	147 ^f^	3.5 ^a^	86 ^c^
CCtrl	66.0 ^abc^	77.7 ^a^	3.21 ^c^	0.48	16 ^ab^	282 ^de^	277 ^gh^	397 ^g^	120 ^e^	4.5 ^ab^	92 ^e^
CEnzP1	65.9 ^ab^	79.5 ^def^	3.21 ^c^	0.56	22 ^de^	269 ^cd^	265 ^f^	374 ^f^	108 ^d^	3.5 ^a^	95 ^g^
CEnzP2	66.9 ^bcde^	78.7 ^abcd^	2.91 ^b^	0.68	21 ^bcde^	302 ^f^	279 ^gh^	361 ^ef^	82 ^b^	23.5 ^c^	94 ^f^
CEnzA	66.2 ^abcd^	80.4 ^fg^	3.41 ^c^	0.56	21 ^bcde^	23.5 ^a^	14 ^a^	19 ^a^	5 ^a^	9.5 ^ab^	0 ^a^
CEnzF1	66.7 ^abcde^	78.4 ^abc^	2.85 ^b^	0.56	18 ^abcd^	292 ^ef^	285 ^h^	390 ^g^	105 ^d^	7 ^ab^	91 ^e^
CEnzP1 + F1	66.0 ^abc^	79.3 ^cde^	3.21 ^c^	0.58	21 ^cde^	260 ^c^	254 ^e^	335 ^d^	81 ^b^	6 ^ab^	95 ^g^
CEnzP1 + Ph	66.0 ^abc^	79.1 ^bcd^	3.25 ^c^	0.56	16 ^ab^	280 ^de^	273 ^fg^	371 ^f^	98 ^c^	7 ^ab^	94 ^f^
CEnzP1 + A	65.3 ^a^	80.8 ^g^	3.39 ^c^	0.62	24 ^e^	25.5 ^a^	15 ^a^	20 ^a^	5 ^a^	10.5 ^b^	0 ^a^
CChem	67.1 ^bcde^	78.3 ^ab^	2.88 ^b^	0.57	16 ^ab^	468 ^j^	309 ^i^	413 ^h^	104 ^d^	159 ^g^	85 ^b^
CLab1	66.5 ^abcde^	78.3 ^ab^	2.52 ^a^	0.51	14 ^a^	334 ^gh^	242 ^d^	326 ^cd^	84 ^b^	92 ^d^	85 ^b^
CLab2	66.8 ^bcde^	78.6 ^abcd^	2.77 ^ab^	0.52	14 ^a^	366 ^i^	272 ^fg^	354 ^e^	82 ^b^	94 ^d^	86 ^c^
CLab3	67.3 ^cde^	78.6 ^abcd^	2.73 ^ab^	0.55	14 ^a^	345 ^h^	248 ^de^	334 ^d^	86 ^b^	97.5 ^d^	86 ^c^
CLab1 + L2	67.5 ^de^	78.7 ^bcd^	2.73 ^ab^	0.63	18 ^abcd^	331 ^g^	226 ^c^	306 ^b^	80 ^b^	105 ^e^	87 ^d^
CLab + Enz	67.8 ^e^	80.2 ^efg^	2.65 ^ab^	0.76	23 ^e^	326 ^g^	213 ^b^	319 ^bc^	105 ^d^	113 ^f^	87 ^d^

Letters denote homogenous subsets in ANOVA analysis.

**Table 6:** Bread quality parameters measured from the breads made with bioprocessed sorghum and cowpea. Sample codes are explained in Table 1.

Samples	Specific Volume (mL/g)	Crumb Moisture (%)	Hardness (N)	Springiness	Cohesiveness	Resilience	Chewiness
Ref	1.51 ± 0.06 ^bcd^	51.1 ± 0.3 ^c^	17.7 ± 1.8 ^ab^	0.884 ± 0.011 ^bcd^	0.591 ± 0.028 ^cd^	0.283 ± 0.018 ^c^	9.2 ± 0.7 ^c^
SCtrl	1.34 ± 0.05 ^ab^	49.5 ± 0.3 ^ab^	38.0 ± 4.3 ^d^	0.890 ± 0.006 ^bcd^	0.585 ± 0.017 ^c^	0.293 ± 0.009 ^cd^	19.8 ± 2.6 ^f^
SEnzP4	1.57 ± 0.03 ^cd^	49.4 ± 0.1 ^ab^	15.9 ± 1.6 ^a^	0.835 ± 0.015 ^b^	0.518 ± 0.021 ^b^	0.224 ± 0.012 ^b^	6.9 ± 0.7 ^ab^
SAlc	1.30 ± 0.08 ^ab^	49.7 ± 0.1 ^b^	30.5 ± 0.9 ^c^	0.912 ± 0.005 ^cd^	0.625 ± 0.011 ^cde^	0.325 ± 0.008 ^ef^	17.3 ± 0.6 ^e^
SLab + EnzMix	1.43 ± 0.04 ^abc^	49.0 ± 0.2 ^a^	21.6 ± 1.3 ^b^	0.922 ± 0.010 ^d^	0.654 ± 0.009 ^e^	0.349 ± 0.007 ^f^	13.0 ± 0.6 ^d^
CCrtl	1.58 ± 0.02 ^cd^	50.6 ± 0.0 ^c^	18.4 ± 2.6 ^ab^	0.852 ± 0.099 ^bc^	0.629 ± 0.034 ^de^	0.315 ± 0.029 ^de^	9.9 ± 1.9 ^c^
CEnzP1 + F1	1.64 ± 0.08 ^de^	50.7 ± 0.1 ^c^	17.2 ± 1.3 ^ab^	0.909 ± 0.010 ^cd^	0.625 ± 0.018 ^cde^	0.315 ± 0.012 ^de^	9.7 ± 0.5 ^c^
CEnzP1 + A	1.78 ± 0.09 ^e^	54.0 ± 0.2 ^e^	37.8 ± 3.3 ^d^	0.531 ± 0.028 ^a^	0.307 ± 0.018 ^a^	0.114 ± 0.008 ^a^	6.2 ± 1.1 ^ab^
CLab1 + L2	1.61 ± 0.05 ^de^	53.3 ± 0.3 ^d^	14.8 ± 1.5 ^a^	0.908 ± 0.013 ^cd^	0.648 ± 0.017 ^e^	0.331 ± 0.012 ^ef^	8.7 ± 0.5 ^bc^
CChem	1.64 ± 0.10 ^de^	50.5 ± 0.1 ^c^	15.8 ± 1.1 ^a^	0.894 ± 0.009 ^bcd^	0.632 ± 0.011 ^de^	0.315 ± 0.009 ^de^	8.9 ± 0.5 ^bc^

Letters denote homogenous subsets in ANOVA analysis.

## Data Availability

Data is contained within the article.
